# Impact of Cholesterol on the Stability of Monomeric and Dimeric Forms of the Translocator Protein TSPO: A Molecular Simulation Study

**DOI:** 10.3390/molecules25184299

**Published:** 2020-09-19

**Authors:** Zeineb Si Chaib, Alessandro Marchetto, Klevia Dishnica, Paolo Carloni, Alejandro Giorgetti, Giulia Rossetti

**Affiliations:** 1 Institute for Neuroscience and Medicine (INM-9) and Institute for Advanced Simulations (IAS-5) “Computational biomedicine”, Forschungszentrum Jülich, 52425 Jülich, Germany; z.si.chaib@fz-juelich.de (Z.S.C.); a.marchetto@fz-juelich.de (A.M.); p.carloni@fz-juelich.de (P.C.); 2 Faculty of Mathematics, Computer Science and Natural Sciences, RWTH Aachen, 52062 Aachen, Germany; 3 Department of Biotechnology, University of Verona, 37134 Verona, Italy; klevia.dishnica@univr.it; 4 Institute for Neuroscience and Medicine (INM-11) “Molecular Neuroscience and Neuroimaging”, Forschungszentrum Jülich, 52425 Jülich, Germany; 5 Jülich Supercomputing Center (JSC), Forschungszentrum Jülich, 52425 Jülich, Germany; 6 Department of Hematology, Oncology, Hemostaseology, and Stem Cell Transplantation University Hospital Aachen, RWTH Aachen University, Pauwelsstraße 30, 52074 Aachen, Germany

**Keywords:** TSPO, Martini force-field, cholesterol

## Abstract

The translocator protein (TSPO) is a transmembrane protein present across the three domains of life. Its functional quaternary structure consists of one or more subunits. In mice, the dimer-to-monomer equilibrium is shifted in vitro towards the monomer by adding cholesterol, a natural component of mammalian membranes. Here, we present a coarse-grained molecular dynamics study on the *mouse* protein in the presence of a physiological content and of an excess of cholesterol. The latter turns out to weaken the interfaces of the dimer by clusterizing mostly at the inter-monomeric space and pushing the contact residues apart. It also increases the compactness and the rigidity of the monomer. These two factors might play a role for the experimentally observed incremented stability of the monomeric form with increased content of cholesterol. Comparison with simulations on bacterial proteins suggests that the effect of cholesterol is much less pronounced for the latter than for the *mouse* protein.

## 1. Introduction

The translocator protein (TSPO), also known as peripheral benzodiazepine receptor, is a membrane protein conserved across the three domains of life [1]. During evolution, the TSPO gene family in bacteria and plants has expanded its roles [2] from an environmental sensor (such as sensitivity to salt stress in cyanobacteria and plants [3]) to a functional bioregulator, for instance facilitating the switch between photosynthesis and respiration, porphyrin transport and regulation of photosynthetic genes in the *Rhodobacter* (*Rs*) bacterium [4].

Mammalian TSPOs are mostly expressed in the outer mitochondrial membrane (OMM) [5]. Their functions may be rather diverse. For instance, in humans, the protein is involved in apoptosis, autophagy, inflammation along with cholesterol and porphyrin transport [6]. The latter is reminiscent of bacterial TSPO functions [7]. Human TSPO has emerged as an important neuropharmaceutical target. Indeed, TSPO levels are upregulated in activated glial cells, especially in microglia during neural disorders or injury [8]. As a result, radioligands binding to human TSPO in positron emission tomography (PET) scan may be used to diagnose CNS disorders [7].

TSPO consists of one or more units of about 18 KDa molecular weight. The topology of each unit, conserved across bacteria and mammals, consists of five transmembrane helices. These are tightly packed together (TM1–TM5) and connected by loops, located (for mammals) in the cytoplasm (LP-I and LP-III) and in the intermembrane space (LP-II and LP-IV) [7]. Structural information on TSPO is available from the bacteria *Bacillus Cereus* (*Bc*) and *Rhodobacter sphaeroides* (*Rs*), as well as from mice.

No monomer unit has been observed for *Rhodobacter sphaeroides* dimer (*Rs*TSPO), neither in vitro nor in vivo [9]. In the *Rs*TSPO X-ray structure, the protein is present as a dimer [9], suggesting that the latter is the minimum functional unit of the protein [10]. The X-ray structure of the protein from *Bacillus Cereus* (*Bc*) has also been solved as a dimer [11], although crystal packing forces might have affected the oligomerization state [12]. In *mouse* TSPO, multimers (up to six units) exist in vivo [13,14] and these different oligomeric states may be associated with different functions [6,15]. The structure of the *mouse* monomer in the presence of detergent micelles has been solved by solution NMR [16].

Cholesterol represents 10% of OMM [17]. In addition, OMM contains phosphatidylcholine (PC, 40%), phosphatidylethanolamine (PE, 38.9%), phosphatidylserine (PS, 14.2%), phosphatidylinositol (PI, 5.9%) and cardiolipin (CDL) 0.8% [17]. Cholesterol has high (nanomolar) affinity for *mouse* TSPO [18] and it affects *mouse* TSPO oligomeric state. It stabilizes in vitro the monomer against the dimer [19], possibly by binding to a conserved “cholesterol-recognition motif” of the protein [18]. This is referred to as “LAF-CARC”. CARC is defined by the linear array of residues (L/V)-X1−5-(Y)-X1−5-(K/R), located in the outer membrane leaflet and its reverse sequence, CRAC, (K/R)-X1−5-(Y/F)-X1−5-(L/V) in the inner one [20,21]. LAF (Leu-Ala-Phe) is a cholesterol binding enhancement motif associated with CRAC located one helix turn above the CRAC domain in mammalian TSPO [22]. Cholesterol is instead absent in the bacterial membrane, and it binds to the bacterial proteins only in the micromolar range [10]. However, bacteria and archaea have a similar CRAC sequence with the central Y replaced by F, probably to adapt Hopanoids, which have a ring structure similar to that of cholesterol, have been identified in bacteria as serving a function analogous to that of cholesterol in the higher animals [23]. In *Rs*TSPO cell membrane, four major classes of phospholipids are present, namely, PE, PC, phosphatidylglycerol (PGL), and CDL, in a ratio of approximately 5:2:2.4:0.6 (PE/PC/PGL/CDL) [24]. In *Bacillus cereus* dimer (*Bc*TSPO), the major lipids that form the membrane are PE 43%, PGL 40% and CDL 17% [25].

Here, we investigate *mouse* and bacterial TSPOs embedded in membranes with different content of cholesterol by using coarse-grained molecular dynamics (MD) simulations based on the Martini force field (version 2.2) [26]. Martini is a widely used coarse-grained force field. Here, several atoms are grouped together in a “virtual” bead that interact through an effective potential [27]. The simulations are performed on *mouse* TSPO monomer and *mouse* TSPO dimers along with *Rs*TSPO and *Bc*TSPO dimers, embedded in a membrane resembling the composition of their respective realistic environment (OMM, *Rs* bacterial membrane (Rs_mem) and *Bc* bacterial membrane (Bc_mem) for *mouse* TSPO, *Rs*TSPO and *Bc*TSPO, respectively, see Table S1). Comparison is then made with models embedded in model membranes with physiological cholesterol content (10%, OMM), high cholesterol (28%, chl_mem) and without cholesterol (Bc_mem and Rs_mem) (Table S1).

**Scheme 1:**
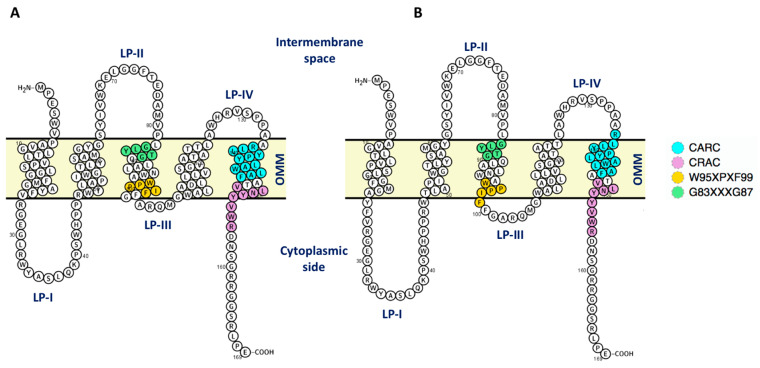
Topology of the NMR *mouse* translocator protein (TSPO) monomer (*m*TSPO_mon) (**A**) and of the monomeric *mouse* TSPO model based on *Rhodobacter sphaeroides* dimer (*Rs*TSPO) structure (*m*TSPO(Rs)_mon) (**B**).

The calculations on the bacterial proteins are based on the corresponding X-ray structures of the dimer [9,11]. Those on *mouse* TSPO are based on two models of the dimer built by us (available at [28]), based on *Rs*TSPO dimer X-ray and *mouse* TSPO monomer NMR structures (see Scheme 1). The first was fairly consistent with experimental data concerning the dimeric interface [19]; the second showed larger discrepancies with experiments. Both models were refined here in an OMM-mimicking membrane by molecular simulation. Notably, the resulting structures were both able to reproduce the experimental dimer interface showing the impact of the membrane environment on structural models of membrane proteins. Models of the *mouse* proteins, embedded in Dodecylphosphocholine (DPC) [29] and 1,2-dimyristoyl-sn-glycero-3-phosphocholine (DMPC)/cholesterol mixtures [30], have been recently reported. Anticipating our results, our simulations suggest that cholesterol may stabilize the monomer by reducing the local and the global fluctuation of the protein; while it might destabilize the dimer by clusterizing at the subunit–subunit interface and push apart the contact residues. The impact of the molecule on the bacterial protein dimers appears to be much less evident.

## 2. Results

### 2.1. TSPO Mouse Dimers

Our previous work presented two models of the protein with and without the nanomolar inhibitor PK-11195 [28]. These models were based on the *Rs*TSPO dimer X-ray structure (PDB accession code 4UC1) and on the NMR *mouse* TSPO monomer structure (PDB accession code 2MGY), in complex with PK-11195 [9,16]. The first, which we call here *m*TSPO(Rs), was obtained by homology modeling on *Rs*TSPO dimer; the second, *m*TSPO, was obtained by duplicating the monomeric *mouse* TSPO NMR structure and guiding the building of the dimer on the experimental chemical shifts in [19]. The proteins without ligands underwent refinement here in two different membrane environments (see Table S1). As shown in the next two sections, the resulting models are consistent with most of the available experimental data, which concern the dimer interface (i.e., the ensemble of contact residues across the two monomers) [19].

#### 2.1.1. Models Refinement

**Figure 1:**
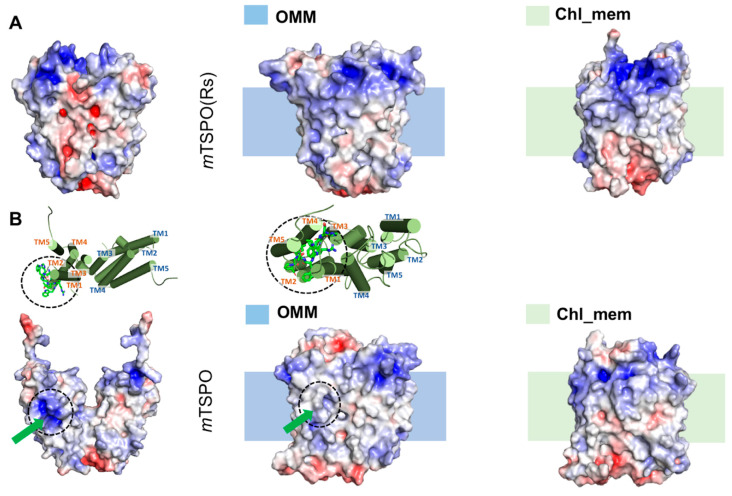
Electrostatic surface potential of the initial [28] and final molecular dynamics (MD) structures of (**A**) dimeric *mouse* TSPO model based on *Rs*TSPO dimer (*m*TSPO(Rs)) and (**B**) dimeric *mouse* TSPO model based on NMR structure (*m*TSPO), in the different membrane environments simulated here. The initial structure is the one prior to the embedding in the model membranes and it is the first structure of each raw image. The shadow behind the second and the third structures of each column indicate the approximate location of the membrane. The upper part “insert” in B indicates how the exposed polar side-chains in *m*TSPO twist toward the bulk of the protein after the equilibration. The green arrows indicate the above-mentioned residues in the protein surface. The MD structures were backmapped to all-atom resolution using the backward.py script [31]. The red and blue surfaces represent negative and positive electrostatic potentials, respectively. The maximum values of the potentials are −5 kT/e and +5kT/e, respectively (where *k* is Boltzmann constant, T is the temperature and e is the electric charge of an electron).

In our previous structural prediction of *m*TSPO(Rs) [28], the G83XXXG87 and the W95XPXF99 dimerization motif were located at the dimer interface, consistently with the experimental data [19]. The monomer–monomer interface turned out to involve V80, G83, W93, I98, and A102 from TM-III and D111, V118 from TM-IV, consistently with NMR data [19] (Table 1). However, a few residues—known to be part of the interface—were not sufficiently close to it, even though they were localized near to it [28]. The protein exhibited an electrostatic profile favorable for membrane embedding (Figure 1A) [28].

Here, we refined the models by embedding the systems in both OMM and chl_mem and performing 8 μs MD with the Martini 2.2 coarse-grained force field [26] (see methods). The chosen model membranes feature physiological (10% of total phospholipids—OMM) and high (28%—chl_mem) cholesterol content (see Table S1 for composition). After ~1 μs, the structure was equilibrated as shown by the Root Mean Square Deviation (RMSD) (Figure S1). The RMSD between the initial and final structure was not very high (4 Å in the TM region), suggesting significant but not very large displacement from the initial position. The structure at the end of the dynamics displayed a compact dimer–dimer interface involving a higher number of residues than the starting structure. These include V80, G83, W93, W95, I98, F100, G101 from TM-III and D111, V118 from TM-IV, reported to be at the interface, accordingly to the NMR data [19] (Table 1).

**Figure 2:**
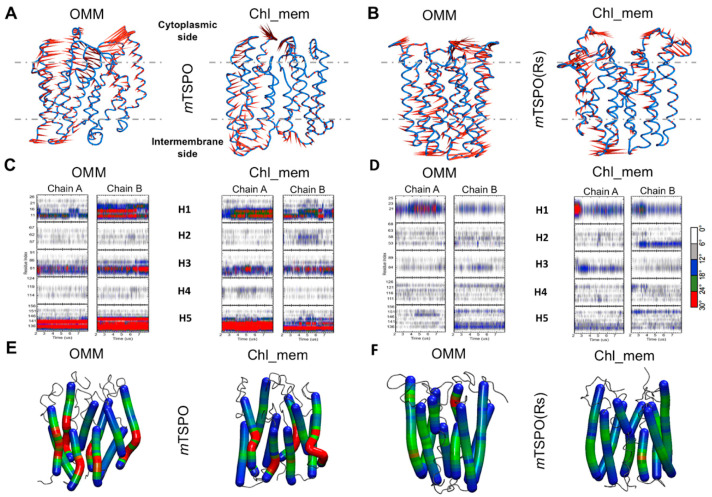
Porcupine plots depicting prominent motions averaged across the first normal mode for (**A**) *m*TSPO, (**B**) *m*TSPO(Rs) embedded in OMM and cholesterol rich membrane (chl_mem); the corresponding plots for the second normal mode are reported in Figure S4. A dotted line marks the approximate location of the membrane. The helix bending of (**C**) *m*TSPO, (**D**) *m*TSPO(Rs) in OMM and chl_mem is plotted as a function of the simulation time. The corresponding helix “hinge” regions are also shown on *m*TSPO (**E**) and *m*TSPO(Rs) (**F**) structures in OMM and chl_mem: the highly bent regions are highlighted in red color, while the least and moderately bent regions are highlighted in blue and green colors, respectively.

The *m*TSPO starting model, built in [28], did not reproduce several of the experimental data: indeed, the residues experimentally identified at the monomer–monomer interface, were separated by a distance of 9 Å or more (N92, W93, W95, I98, F100, G101, and A102, Table 1) [28]; in addition, several charged residues were exposed in the transmembrane region, not allowing for a favorable embedding in the membrane [28]. Here, the refinement by MD leads large conformational rearrangements: the helices tend to twist to bury the exposed polar residues toward the core of the bundle (Figure 1B), while in parallel, the two monomers tend to get closer to each other to maximize the contact between residues at the interface (Table 1). Helices one, three and five significantly bend in the intermembrane region of the receptor (Figure 2A,C,E).

The structures were equilibrated after ~1 μs and ~2 μs in OMM and in chl_mem, respectively, as shown by the plot of RMSDs as a function of the simulated time (Figure S1). Notably, the MD structures at the end of the dynamics showed similar monomer–monomer interface’s features as *m*TSPO(Rs) equilibrated structure (Table 1) and a favourable electrostatic profile for membrane embedding.

#### 2.1.2. Structural Properties of the Models

We present here the differences in structure and conformational fluctuations of *m*TSPO and *m*TSPO(Rs) dimers during the equilibrated trajectory, namely for the last 6 μs.

The RMSDs (Figure S1 and Table 2), the Root Mean Square Fluctuations (RMSFs) (Figures S2A,B and S3) and the gyration radii (Figure S2C,D and Table 2) of the two models do not vary significantly by changing membrane environment (Figures S1 and S2). High flexibility regions are observed for the loops and the termini, as expected (Figure S2).

**Figure 3:**
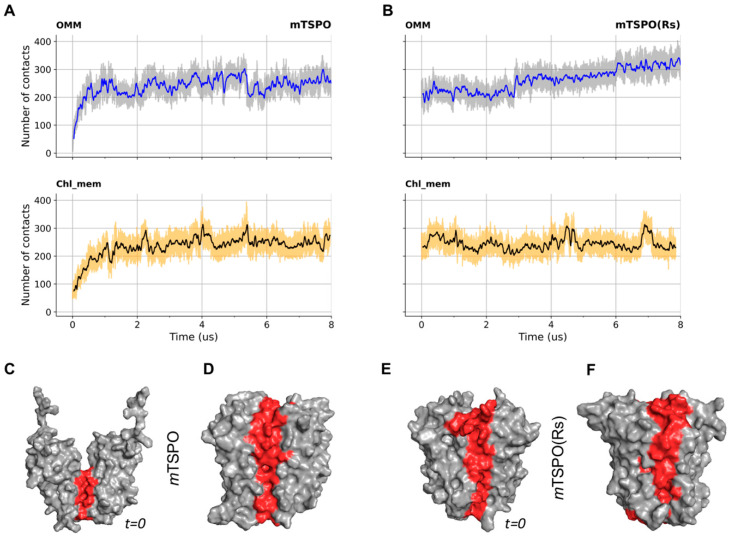
Number of monomer–monomer contact beads as a function of the simulation time in OMM and chl_mem for (**A**) *m*TSPO, (**B**) *m*TSPO(Rs). A cutoff of 0.6 nm was used. Surface representation of *m*TSPO structures before (**C**) and after (**D**) equilibration in OMM, as well as *m*TSPO(Rs) structures before (**E**) and after (**F**) equilibration in OMM, are shown. Their surface is represented in grey color, except for the residues in contact at the dimer interface which are highlighted in red.

A principal component analysis (PCA) was used to identify differences in large scale motions of the proteins (Figure S3). We represent the first two eigenvectors (which cover between 31% and 38% of the motions of the proteins) as porcupine plots to show the direction and magnitude of selected eigenvectors for each of the backbone beads. *m*TSPO’s conformational flexibility was larger than that of *m*TSPO(Rs) in OMM (Figure 2A), as can be seen by the porcupine plots of the first and second eigenvector (Figure S4). The flexibilities were similar for the two proteins embedded in chl_mem (Figure 2 and Figure S4), however, at the dimer interface of both dimers, the two first eigenvectors “push” the residues far apart, hinting toward a destabilization of the dimers’ interface (Figure 2 and Figure S4). Helix conformational changes were calculated using the “Bendix” software v.1.1 [32]. This software uses a sliding window of four residues to give local helix axes that are joined by a spline. Analysis is performed on this axis to give local angles along the length of the helix versus time. The helix distortion distribution is visualized across residues by heatmap color-coding according to local angle magnitude, which highlights nonlinear behaviour of the alpha helix axes. We noticed that in *m*TSPO(Rs), the axes of the helices are mostly linear, while in *m*TSPO, TM1, TM3 and TM5 are bent in the lower part corresponding to the mitochondria side (Figure 2C,E) in both OMM and chl_mem. The latter behaviour highlights the main difference in the geometry of the helix bundle on passing from *m*TSPO to *m*TSPO(Rs). The number of contacts at the dimer interface decreases in chl_mem (Figure 3).

### 2.2. Mouse TSPO Monomers

#### 2.2.1. Models Refinement

We predicted here the monomer structures based on the *Rs*TSPO structure [9] (*m*TSPO(Rs)_mon hereafter) and the NMR structure of monomeric *mouse* TSPO [16] (*m*TSPO_mon). The electrostatic surface of *m*TSPO(Rs)_mon starting model was favorable to membrane embedding, while this was not the case for *m*TSPO_mon [28], similarly to what observed for the initial structures of the corresponding dimers. The proteins were embedded in OMM and in chl_mem. They underwent 8 μs MD with the Martini coarse-grained force field version 2.2 [26], as in the case of the dimers.

**Figure 4:**
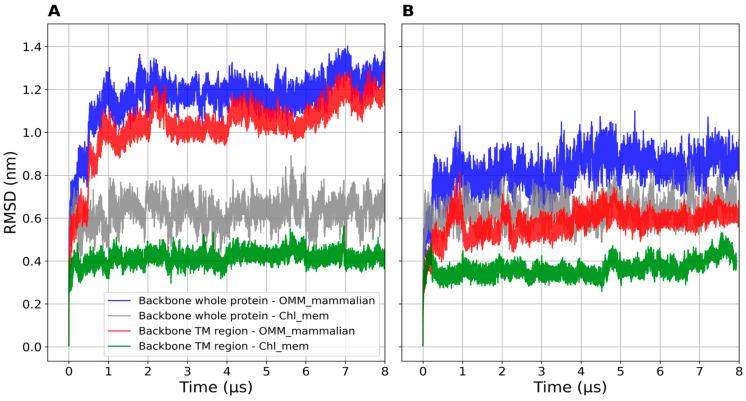
Backbone beads RMSD of (**A**) *m*TSPO_mon and (**B**) *m*TSPO(Rs)_mon (whole protein and TM regions) embedded in OMM and chl_mem plotted as a function of the simulated time. The RMSDs are computed with respect to the initial structures.

**Figure 5:**
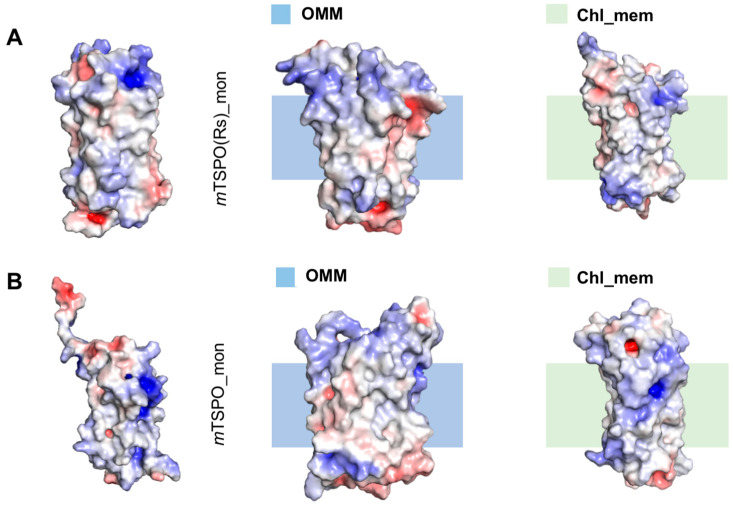
Electrostatic surface potential of the initial and equilibrated conformations, extracted from the trajectories after convergence (7.5 μs) and backmapped to all-atom resolution with the amber force field [33] using backward.py [31] of *m*TSPO(Rs)_mon (**A**) and *m*TSPO_mon (**B**) embedded in OMM and chl_mem. Red and blue surfaces represent negative and positive electrostatic potentials (−5 kT/e, +5kT/e) respectively.

*m*TSPO(Rs)_mon turned out to be equilibrated after about 1 μs, as shown by the plot of the RMSD as a function of simulated time (Figure 4). The electrostatic surface of the protein already showed a favorable distribution of apolar/polar residues for membrane embedding (Figure 5A). Further optimization of side chain distribution can be observed after equilibration (Figure 5A).

*m*TSPO_mon required instead 2 μs to be equilibrated (Figure 4). It indeed underwent several conformational rearrangements similarly to what happened for the corresponding dimer. As a result, the protein becomes more compact. In addition, the electrostatic surface of the TM region in the equilibrated structures exposed more hydrophobic residues with respect to the initial structure. This rendered the protein compatible with the embedding in the membrane environment (Figure 5B), in contrast to the initial NMR structure [28].

#### 2.2.2. Structural Features

Here we present the differences in structure and conformational fluctuations of *m*TSPO_mon and *m*TSPO(Rs)_mon during the equilibrated trajectory, namely for the last 6 μs.

**Figure 6:**
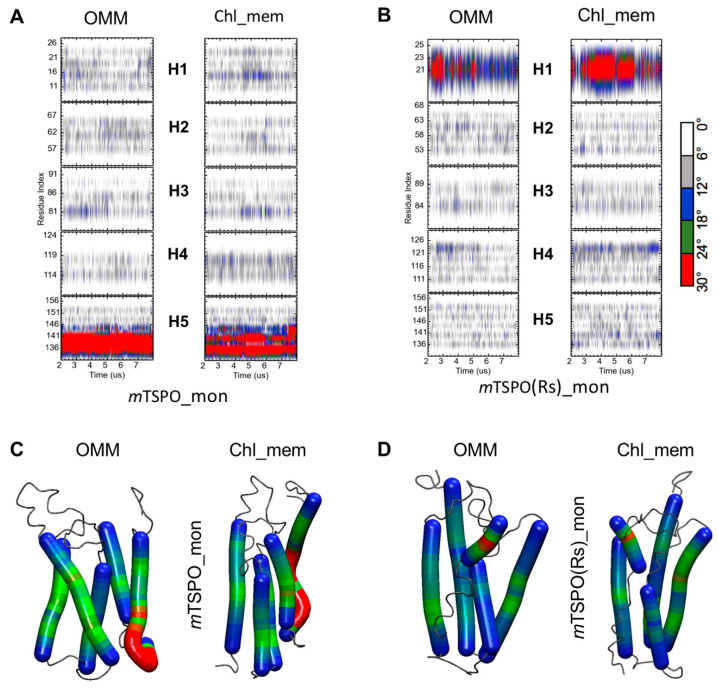
The helix bending of (**A**) *m*TSPO_mon and (**B**) *m*TSPO(Rs)_mon embedded in both OMM and chl_mem as a function of the simulation time. Only the last 6 μs are shown. The corresponding helix “hinge” regions are also shown on *m*TSPO_mon (**C**) and *m*TSPO(Rs)_mon (**D**) structures in OMM and chl_mem: the highly bent regions are highlighted in red color, while the least and moderately bent regions are highlighted in blue and green colors, respectively.

The two models differ significantly: *m*TSPO_mon features a kink in helix five (Figure 6A), while *m*TSPO(Rs)_mon has a kink in helix one (Figure 6B). The averaged backbone beads RMSDs decrease in all circumstances with cholesterol content (Figure 4 and Table 2). Instead, the radii of gyration are rather similar (Figure S5). The averaged RMSF values decrease in correspondence with the transmembrane regions (TM1: 7–26, TM2: 46–63, TM3: 82–101, TM4: 106–124 and TM5: 134–153 for *m*TSPO and TM1: 8–23, TM2: 48–62, TM3: 83–98, TM4: 107–123 and TM5: 136–152 for *m*TSPO(Rs)) on passing from the systems embedded in OMM to chl_mem (Figure S6). High flexibility regions are observed in the loops and terminal regions, as expected (see Figure S6). The region 125–140 was more flexible in *m*TSPO_mon than in *m*TSPO(Rs)_mon, which harbors the cholesterol-binding motif [19].

**Figure 7:**
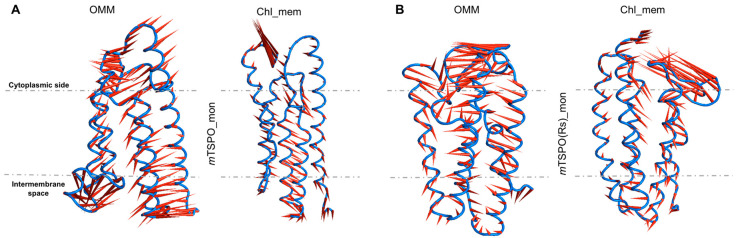
Porcupine plots depicting prominent motions averaged across the first normal mode for (**A**) *m*TSPO_mon and for (**B**) *m*TSPO(Rs)_mon, in both OMM and chl_mem. A dotted line marks the approximate location of the membrane. Longer spines indicate the region with higher fluctuations for the first normal mode. These correspond to the cytoplasmic loops. The analysis was performed on the equilibrated part of the trajectory. The second normal mode is reported in Figure S8.

The first three eigenvectors in the PCA occupy between 35.8% and 50.4% of the conformational space of the proteins (Figure S7). They are larger for the proteins embedded in OMM than those embedded in the chl_mem (Figure 7 and Figure S8). Therefore, we suggest that the binding of cholesterol reduces the conformational fluctuations of the proteins.

### 2.3. Cholesterol Occupancy

**Figure 8:**
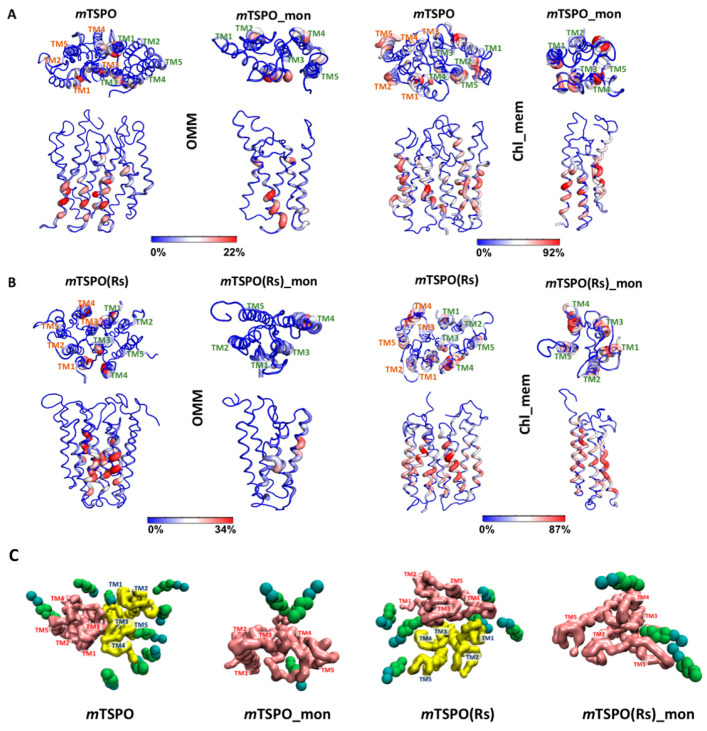
Cholesterol occupancy (i.e., percentage of frames where the lipid is in contact with a given residue (0–100%)) for (**A**) *m*TSPO, *m*TSPO_mon and (**B**) *m*TSPO(Rs), *m*TSPO(Rs)_mon in both OMM and chl_mem. The analysis was performed on the equilibrated part of the trajectory, that is, the last 6 μs. Labeling of the helices is done only on the top view of each system for clarity. (**C**) Selected snapshots from our simulations illustrating cholesterol position at the protein surface for *m*TSPO, *m*TSPO_mon, *m*TSPO(Rs) and *m*TSPO(Rs)_mon in OMM membrane.

Given the significant effects of cholesterol described above, to conclude, we analyzed the occupancy of cholesterol molecules in the two different membrane environments, for both monomer and dimer *mouse* TSPO models. In an overabundance of cholesterol (that is, for the proteins embedded in chl_mem), cholesterol molecules are spread rather homogeneously around the TM regions (Figure 8). This is fully consistent with the coarse-grained dynamics simulation study by Rao et al. [30] on the same protein, performed in a DMPC/cholesterol mixture. These authors found that “cholesterol molecules are spread homogeneously in the membrane despite the presence of dimers” [30]. In contrast, for the proteins embedded in a membrane with physiological content of cholesterol (OMM), cholesterol clusters at TM1, TM3 and TM4 located at the dimer interface (Figure 8), and, for the *m*TSPO_mon, also at TM5. This helix contains the two well-known cholesterol-binding motifs, CRAC (residues 149–156) and its reverse counterpart CARC (residues 135–146) [20,21].

### 2.4. Bacterial TSPO Dimers

*Bc*TSPO and *Rs*TSPO embedded in the Bc_mem and Rs_mem (without cholesterol, as in natural bacterial membranes [25,34] respectively, and chl_mem (with 28% cholesterol) (Table S1) underwent 2 μs coarse-grained MD simulations. As shown by the RMSD plot as a function of simulated time (Figure S9), the systems reached equilibrium already after 700 ns, possibly because in this case we started from X-ray structures and not theoretical models. The average displacement from the initial structures were ~0.6 nm for both structures (Figure S9).

**Figure 9:**
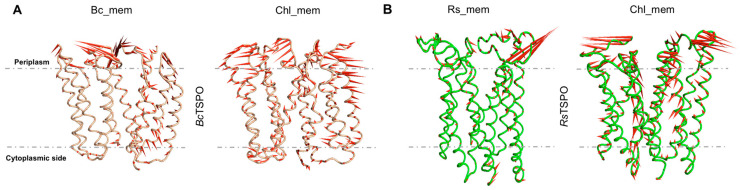
Porcupine plots depicting prominent motions averaged across the first normal mode for: (**A**) *Bc*TSPO in Bc_mem and chl_mem and (**B**) *Rs*TSPO in Rs_mem and chl_mem, respectively. A dotted line marks the approximate location of the membrane. Longer spines indicate the region with higher fluctuations for the first normal mode. These correspond to the periplasm loops. The analysis was performed on the equilibrated part of the trajectory, namely the last 1.3 μs.

The RMSD, RMSF and gyration radius (Figures S9–S11) as well as the calculated electrostatic surfaces along the simulation (Figure S12) were similar across the four systems investigated here. Additionally, the bending of the helices does not change on passing from the bacterial membranes to chl_mem (Figure S15). The contacts at the interface of the proteins embedded in Bc_mem and Rs_mem are larger than those in chl_mem (Figure S13). In our PCA analysis (Figure S14), the first two eigenvectors exhibit very small fluctuations at the interface in both membranes (Figure 9). Based on all of these observations, we conclude that the presence of cholesterol impacts on the structural determinants and conformational fluctuations of the proteins to a lesser extent than in the *mouse* proteins.

## 3. Discussion

Cholesterol is an essential component of mammalian membranes [35]. Among the TSPO functions gained during speciation and evolution leading to mammals, is the binding and trafficking of this molecule [1]. Recent work by Jaipura et al. has shown that cholesterol stabilizes the monomeric against the dimeric state in *mouse* TSPO [19]. Here, we present a coarse-grained MD study on these proteins embedded in membranes at different cholesterol contents to attain an insight on this intriguing effect of this molecule. Comparison is made with bacterial proteins, suggested to exist in dimeric form and binding with much less affinity cholesterol than *mouse* TSPO.

Dimer and monomer models, built by us in [28] and refined here by coarse-grained MD, were embedded in two types of membranes: (i) OMM, which contains six different types of lipids and around 10% of cholesterol. This provides a quasi in-vivo mimetic membrane environment; (ii) chl_mem, with a high cholesterol content (28% instead of 10%).

The dimeric models were fully consistent with the experimental data, which are available only for this state [19] (Table 1). Besides, our model based on the *mouse* NMR monomer structure [16], exhibits a similar interface to that of a recent dimeric model presented [30] involving TM1 and TM3 helices. The electrostatic potential surfaces of dimer and monomer initial models based on *Rs* bacterial structure is suitable for embedding in the cell membrane. This contrasts with the *mouse* monomeric NMR structure [16] (used here as a starting model for some of our simulations). The electrostatic potential surface is favorable for membrane embedding only after equilibration of the protein in a realistic membrane environment. This supports the hypothesis that the ionic detergents used in the structure determination experiment may have altered some key structural features relevant for the stability of the protein in a membrane environment [12,29].

Both the simulations of the monomers and the dimers hint a stabilization of the latter by adding cholesterol. First, at the physiological content of cholesterol, this is located mostly at residues at the dimeric interface, while in *m*TSPO_mon, cholesterol is found at the two cholesterol-binding motifs, CRAC and CARC [20,21]; second, an overabundance of cholesterol causes a decrease in subunit–subunit contacts in the dimer: consistently, the large-scale motions might favor a detachment of the two units by pushing apart the residues in contact at the interface, as shown by our PCA analyses. All these features point to a destabilizing effect of cholesterol on the dimeric structure. Finally, under the reasonable assumption that the compactness and rigidity of proteins may correlate with thermodynamic stability [36], one can suggest that cholesterol stabilizes the monomer. Indeed, (i) the size of the monomeric *mouse* TSPO models, in terms of RMSD and gyration radii, decreases with cholesterol content, in contrast to what happens in the dimeric forms; (ii) the fluctuations, as measured by RSMF values, are lower; (iii) the large-scale motions exhibit a smaller amplitude in the presence of cholesterol for both the monomeric proteins.

Cholesterol affects to a lower extent the size and rigidity of the corresponding bacterial proteins. These are naturally embedded in a cholesterol-free membrane and present most probably as dimers [9]. Indeed, RMSD gyration radii do not vary significantly upon the addition of cholesterol. In addition, RMSF and large-scale motion are not significantly affected by the presence of this molecule. However, the contacts at the subunit–subunit interface decrease with an excess of cholesterol (albeit to a smaller extent than what was observed in the *mouse* protein). Hence, the study for the bacterial proteins is not conclusive. This is probably due also to the fact that the CRAC motifs on the bacterial structures (with the central Y replaced by F) were possibly optimized for Hopanoids, that in bacteria can perform a function analogous to that of cholesterol in the higher animals [23].

Are cholesterol binding, TSPO oligomerization state and the TSPO involvement in cholesterol transport or regulation connected among each other? Cholesterol binds to TSPO in vitro and in vivo [18,19]. However, on one hand, cholesterol molecules were identified in none of the reported TSPO X-ray or NMR structures [7]; in addition, no evident channel emerges from a structural analysis of the monomeric or dimeric structures [37]; Finally the dimer interface does not involve any of the residues defined in the CRAC site or the enhancement motif. On the other hand, experiments in mammalian cell culture systems provide evidence of a 800-kDa protein-complex driving cholesterol import, trafficking, and metabolism [15]. In this complex, TSPO is proposed to be the cholesterol binding/sequestering site, along with StAR, and the voltage-dependent anion channel (VDAC). The latter is proposed to be the direct binding partner of TSPO that facilitates transport. This has led to the general suggestion that an “external pathway” allows for cholesterol binding. This pathway is expected to involve a dimer of dimers and/or other binding partner(s) [7,9]. Identifying the hitherto unknown binding interface of TSPO with VDAC and possibly other components of the transduceosome and metabolon complexes is therefore crucial to get further insight on the overall cholesterol transport activity.

## 4. Materials and Methods

### 4.1. System Setup of Coarse-Grained Models

The structure of *mouse* TSPO (*m*TSPO_mon) was the NMR structure (PDB accession code: 2MGY). The first conformation was chosen as it corresponds to the best representative conformer in the ensemble and the ligand PK-11195 was removed. The dimer *m*TSPO was constructed based on the NMR study reported in [16]. Another model of *mouse* TSPO was built by us (*m*TSPO(Rs)_mon and *m*TSPO(Rs) for monomeric and dimeric forms, respectively), by homology modeling, based on the X-ray coordinates of the prokaryotic *Rs*TSPO (PDB accession code: 4UC1) [9].

The structures of bacterial TSPO were taken from the X-ray structures 4RYI (*Bc*TSPO) [11] and 4UC3 (*Rs*TSPO) [9]. Missing residues of 4UC3 between GLU29 and ASN40 in chains A and B were modelled using as template the structure 4UC2 [9], while those between MET147 and ARG157 of chain B were modelled using the same region in chain A of 4UC3 [9]. The Modeller program version 9.19 [38] was used to build the missing regions and the best model according to the Discrete Optimized Protein Energy (DOPE) [39] and GA341 [40] score was selected. The default protonation states at pH 7 were used for the ionizable residues. Protein structures were converted to coarse-grained Martini representations using the martinize.py script [26]. The coarse-grained protein coordinates were then positioned in the center of a simulation box of size 14 × 14 × 16 nm^3^ with its principal transmembrane axis aligned parallel to the z-axis and embedded in different symmetric lipid bilayers: (i) chl_mem contains: phosphatidylcholine (POPC, 31%), phosphatidylethanolamine (POPE, 41%) and cholesterol (CHOL, 28%). (ii) A realistic mammalian outer mitochondrial membrane (OMM) composed of six different lipid species [17,41,42,43,44]: POPC, 40%, 1-stearoyl-2-docosahexaenoyl-phosphatidylethanolamine (SDPE, 38.9%), 1-stearoyl-2-docosahexaenoyl-phosphatidylserine (SDPS, 14.2%), stearoyl-arachidonoyl phosphatidylinositol (SAPI, 5.9%), doubly deprotonated cardiolipin (CDL2, 0.8%) and CHOL, 10% of total phospholipids, providing an in vivo-mimetic membrane environment. (iii) Rs_mem composed of POPC, 20%, POPE, 50%, palmitoyl oleoyl phosphatidylglycerol (POPG, 24%) and cardiolipin (CDL2, 6%) [34] and (iv) Bc_mem containing POPE, 43%, POPG, 40% and CDL2, 17% [25]. NaCl (0.15 M) was added to reach the physiological ions concentration and extra ions were added to neutralize the systems.

### 4.2. Coarse-Grained Simulation Parameters

The Martini coarse-grained force field version 2.2 [26] was used for protein and version 2.0 for lipids. All the simulations were performed using Gromacs 2019.3 [45]. The non-biased simulations were run in the isothermal-isobaric NPT ensemble equilibrium simulations. The temperature was controlled at 315K using V-rescale thermostat [46] with a coupling constant of τ_t_ = 1.0 ps. The pressure was semi-isotropically controlled (i.e., independently in the xy plane and z axis direction) by a Parrinello–Rahman barostat [47] at a reference of p = 1 bar with a coupling constant of τ_p_ = 12.0 ps and compressibility of 3 × 10^−4^. Non-bonded interactions were used in their shifted form with electrostatic interactions shifted to zero in the range of 0–1.1 nm and Lennard-Jones interaction shifted to zero in the range of 0.9–1.1 nm. A time step of 20 fs was used with neighbor lists updated every 20 steps. Periodic boundary condition was used in the x, y and z axis. Data (~8 μs and 2 μs) were collected for *mouse* TSPO and bacterial TSPO simulations, respectively. An overview of the simulations is listed in Table S1.

Analysis of molecular dynamics results. The root mean square deviation of backbone beads (RMSD), the root mean square fluctuations (RMSF) and the radius of gyration (RGYR) were calculated using gmx rms, rmsf and gyrate modules from the Gromacs package [45], respectively. Principal component analysis (PCA) was restricted to backbone beads, as it is less perturbed by statistical noise and provides significant characterization of the essential space motions [48]. It was performed using Gromacs tools gmx covar and gmx anaeig [45]. To visualize the direction and extent of the principal motions of the simulated systems, porcupine plot analysis was performed using modevectors script in Pymol [49]. When applied, for each simulated system, the initial structure was used as a reference. The helix bundle geometrical features were characterized by implementing the Bendix plug-in [32] in VMD software [50], that calculates and visualizes the helix axis evolution over time. It was performed using default parameters for coarse-grained systems. Electrostatic calculations were performed on the initial structures and the ones after equilibrium. Conformations were first extracted and backmapped to all-atom resolution in amber force field [33] using backward.py [31]. Electrostatics were then performed with the APBS Electrostatics plugin in Pymol [51] using default parameters.

The gmx mindist module in Gromacs [45] was used to compute the number of contacts in time between each monomer in the simulated dimers, using the default distance cutoff of 0.6 nm. The cholesterol occupancy (i.e., percentage of frames where lipid is in contact with the given residue (0–100%)) was calculated using the toolkit PyLipID [52] and a distance cutoff of 0.55 and 1.0 nm.

## 5. Conclusions

We have presented a molecular dynamics study on *mouse* TSPO monomeric and dimeric structures. Our models are fully consistent with the available experimental data [19]. In addition, they provide hints on why cholesterol shifts the monomer–dimer equilibrium toward the monomer in *mouse* TSPO. Simulations with the correspondent proteins from *Bc* and *Rs* bacteria might suggest that the destabilization of the dimer by cholesterol (a molecule not present in bacterial membranes) is not as pronounced as in the case of the *mouse* proteins.

How the effect of cholesterol on *mouse* TSPO may affect its function is still unclear. Until recently, there has been general agreement as to the role of TSPO in steroidogenesis. However, recent studies involving genetic depletion of TSPO in mice have created controversy about the role of this protein in steroid and heme synthesis [53]. The discrepant finding in the literature was attributed to the fact that mitochondrial functions are a critical component of cellular survival; the presence of redundancy in mechanisms that mediate crucial mitochondrial functions would be expected [54]. Redundancy in the mitochondrial carrier system transporting small molecules between mitochondria and the cytoplasm was indeed already shown and loss of one could be replaced by another gene or genetic network [55].

Up to now, no human has been identified who lacks TSPO. However, the presence of rs6971 polymorphism (A147T) on the TSPO gene can affect the hypothalamic–pituitary–adrenal axis, predisposing carriers to psychiatric disorders [56]. Therefore, there has been general agreement on the therapeutic value of TSPO as a target for brain pathologies and potentially in conditions of hypogonadism. Although TSPO is a 3.5-billion-year-old protein, we still have unsolved issues concerning its structure, function and pharmacology. Solving these will allow us to better understand not only the evolution of a protein, but also of related processes, such as steroidogenesis, that are critical for life.

## Figures and Tables

**Table 1:** Residues of *mouse* TSPO located at the dimer interface identified experimentally [19] (left), predicted by *m*TSPO (center) and *m*TSPO(Rs) structures at the end of the dynamics.

Region	Inferred by Experiment	*m*TSPO	*m*TSPO(Rs)
Dimer interface	V80, G83, Q88, N92, W93, W95, I98, F100, G101, A102, D111, V118	Initial structures	
		F74, T75, E76, D77, M79, V80, P81, G83, L84, T86, G87, Q88, A90, L91	V6, P7, G10, L11, L13, V14, L17, G18, F20, M21, Y24 V26, R27, M79, V80, L82, G83, L84, Y85, T86, G87, A90, L91, W93, A94, P97, I98, A102, Q104, W107, A108, A110, D111, L114, V118, A121, A125
		Structures after equilibration in outer mitochondrial membrane (OMM)	
		M1, P2, S4, W5, P7, A8, G10, L11, T12, L13, V14, P15, L17, G18, M21, G22, F25, V26, E29, Y34, L37, K39, P40, R46, V67, W68, E70, L71, D77, A78, M79, V80, P81, L82, G83, Y85, T86, G87, Q88, L89, A90, L91, N92, W93, A94, W95, P96, P97, I98, F99, F100, G101, A102, R103, Q104, M105, G106, W107, A108, L109, A110, D111, L113, L114, V115, S116, G117, V118, A121, T122, L124, A125, W126, R128, V129, S130, P131, N151, R166, L167, A168, E169	S4, V6, P7, V9, G10, L11, T12, L13, V14, P15, L17, G18, F20, M21, G22, Y24, F25, V26, R27, G28, E29, G30, L31, R32, W33, W47, W53, Y57, M60, S64, Y65, V67, W68, K69, E70, L71, G72, G73, F74, T75, E76, A78, M79, V80, P81, L82, G83, L84, Y85, T86, G87, L89, A90, L91, W93, A94, W95, P96, P97, I98, F100, G101, R103, Q104, M105, G106, W107, A110, D111, L114, V118, A121, W126

Residues at the interface according to both experiment and simulations are in red and bold.

**Table 2:** Root mean square deviation (RMSD) and radii of gyration values of the systems investigated here, averaged over the equilibrated trajectories (namely the last 6 μs for *mouse* monomeric and dimeric systems and 1.3 μs for the bacterial systems).

System	RMSD (nm)	Radius of Gyration (nm)
mTSPO_mon, OMM	1.2 ± 0.05	1.7 ± 0.03
mTSPO_mon, chl_mem	0.6 ± 0.05	1.8 ± 0.03
*m*TSPO(Rs)_mon, OMM	0.8 ± 0.05	1.7 ± 0.02
*m*TSPO(Rs)_mon, chl_mem	0.6 ± 0.05	1.7 ± 0.02
mTSPO, chain A, OMM	1.1 ± 0.05	1.7 ± 0.02
mTSPO, chain B, OMM	1.0 ± 0.05	1.8 ± 0.02
*m*TSPO, chain A, chl_mem	1.2 ± 0.04	1.7 ± 0.01
*m*TSPO, chain B, chl_mem	1.1 ± 0.06	1.8 ± 0.02
*m*TSPO(Rs), chain A, OMM	0.8 ± 0.05	1.8 ± 0.02
*m*TSPO(Rs), chain B, OMM	0.6 ± 0.05	1.7 ± 0.01
*m*TSPO(Rs), chain A, chl_mem	0.6 ± 0.04	1.7 ± 0.02
*m*TSPO(Rs), chain B, chl_mem	0.5 ± 0.05	1.7 ± 0.01
*Bc*TSPO, chain A, Bc_mem	0.6 ± 0.01	1.6 ± 0.01
*Bc*TSPO, chain B, Bc_mem	0.6 ± 0.02	1.6 ± 0.01
*Bc*TSPO, chain A, chl_mem	0.5 ± 0.03	1.6 ± 0.01
*Bc*TSPO, chain B, chl_mem	0.6 ± 0.02	1.6 ± 0.01
*Rs*TSPO, chain A, Rs_mem	0.7 ± 0.04	1.7 ± 0.01
*Rs*TSPO, chain B, Rs_mem	0.5 ± 0.02	1.7 ± 0.01
*Rs*TSPO_chain A, chl_mem	0.6 ± 0.04	1.7 ± 0.01
*Rs*TSPO_chain B, chl_mem	0.6 ± 0.03	1.7 ± 0.02

## Supplementary Materials

The following are available online. Table S1: Overview of the simulated systems, Figure S1: *m*TSPO and *m*TSPO(Rs) backbone RMSD, Figure S2: RMSF and radii of gyration plots of *m*TSPO and *m*TSPO(Rs), Figure S3: Projection of the first three eigenvectors of *m*TSPO and *m*TSPO(Rs), Figure S4: Porcupine plots depicting prominent motions averaged across the second normal mode for *m*TSPO and *m*TSPO(Rs), Figure S5: Radii of gyration of *m*TSPO_mon and *m*TSPO(Rs)_mon, Figure S6: RMSF plots of *m*TSPO_mon and *m*TSPO(Rs)_mon, Figure S7: Projection of the backbone beads trajectory along the first three eigenvectors for *m*TSPO_mon and *m*TSPO(Rs)_mon, Figure S8: Porcupine plots depicting prominent motions averaged across the second normal mode for *m*TSPO_mon and *m*TSPO(Rs)_mon, Figure S9: Backbone beads RMSD of the whole protein and TM regions for *Bc*TSPO and *Rs*TSPO, Figure S10: RMSF and radii of gyration plots for *Bc*TSPO and *Rs*TSPO, Figure S11: Projection of the first three eigenvectors for *Bc*TSPO and *Rs*TSPO. Figure S12: Electrostatic surface potentials in the initial and final MD structures for *Rs*TSPO and *Bc*TSPO, Figure S13: Number of subunit–subunit contacts plotted as a function of the simulation time for *Bc*TSPO and *Rs*TSPO, Figure S14: Porcupine plots depicting large scale motions averaged across the second normal mode for *Bc*TSPO and *Rs*TSPO, Figure S15: Helix bending of *Bc*TSPO and for *Rs*TSPO.

## Abbreviations

*m*TSPO	Dimeric *mouse* TSPO model based on NMR structure (PDBiD: 2MGY)
*m*TSPO_mon	Monomeric *mouse* TSPO model based on NMR structure (PDBiD: 2MGY)
*m*TSPO(Rs)	Dimeric *mouse* TSPO model based on *Rs*TSPO dimer (PDBiD: 4UC1)
*m*TSPO(Rs)_mon	Monomeric *mouse* TSPO model based on *Rs*TSPO dimer (PDBiD: 4UC1)
*Rs*TSPO	Rhodobacter sphaeroides dimer (PDBiD: 4UC3)
*Bc*TSPO	*Bacillus cereus* dimer (PDBiD: 4RYI)
OMM	Mammalian Outer Mitochondrial Membrane
Chl_mem	Cholesterol rich membrane
*Rs*_mem	*Rs* bacterial membrane
*Bc*_mem	*Bc* bacterial membrane
