# Supplementary material for: Impact of Cholesterol on the Stability of Monomeric and Dimeric Forms of the Translocator Protein TSPO: A Molecular Simulation Study
Source: Molecules. 2020 Sep 19;25(18):4299. doi: 10.3390/molecules25184299 (PMC7570527; doi:10.3390/molecules25184299)
Supplement: Supplementary file 1 [file molecules-25-04299-s001.pdf]

Impact of cholesterol on the stability of monomeric and dimeric forms of the translocator protein TSPO: a molecular simulation study

Zeineb Si Chaib[1,2,*], Alessandro Marchetto[1,3,*], Klevia Dishnica[3], Paolo Carloni[1,2,4], Alejandro Giorgetti[1,3,#], Giulia Rossetti[1,5,6#]

1 Institute for Neuroscience and Medicine (INM-9) and Institute for Advanced Simulations (IAS-5) "Computational biomedicine", Forschungszentrum Jülich, 52425 Jülich, Germany.
2 Faculty of Mathematics, Computer Science and Natural Sciences, RWTH Aachen, 52062 Aachen, Germany
3 Department of Biotechnology, University of Verona, 37134 Verona, Italy
4 Institute for Neuroscience and Medicine (INM-11)"Molecular Neuroscience and Neuroimaging", Forschungszentrum Jülich, 52425 Jülich
5 Jülich Supercomputing Center (JSC), Forschungszentrum Jülich, 52425 Jülich, Germany
6 Department of Oncology, Hematology, Oncology, Hemostaseology, and Stem Cell Transplantation University Hospital Aachen, RWTH Aachen University, Pauwelsstraße 30, 52074 Aachen, Germany.

# Correspondence: g.rossetti@fz-juelich.de (G.R), a.giorgetti@fz-juelich.de (A.G.)
* Equally contributed to this work

Supporting Information

Table S1: Overview of the simulated systems.

Simulation	Protein Structure	Bilayer composition	Duration of the MD
*m*TSPO and *m*TSPO_mon	2MGY	**OMM:** 40% POPC, 38.9% SDPE, 14.2% SDPS, 5.9% SAPI, 0.8% CDL2, and CHOL, 10% of total phospholipids	8 µs
		**Chl_mem:** 31% POPC, 41% POPE and 28% CHOL.	8 µs
*m*TSPO(Rs) and *m*TSPO(Rs)_mon	Homology model based on 4UC1	**OMM:** 40% POPC, 38.9% SDPE, 14.2% SDPS, 5.9%, SAPI, 0.8% CDL2, and CHOL, 10% of total phospholipids)	8 µs
		**Chl_mem:** 31% POPC, 41% POPE and 28% CHOL.	8 µs
*Rs*TSPO	4UC3	**Rs_mem:** 20% POPC, 50% POPE, 24% POPG, 6% CDL2	2 µs
		**Chl_mem:** 31% POPC, 41% POPE and 28% CHOL.	2 µs
*Bc*TSPO	4RYI	**Bc_mem:** 43%POPE, 40%POPG, 17% CDL2	2 µs
		**Chl_mem:** 31% POPC, 41% POPE and 28% CHOL.	2 µs

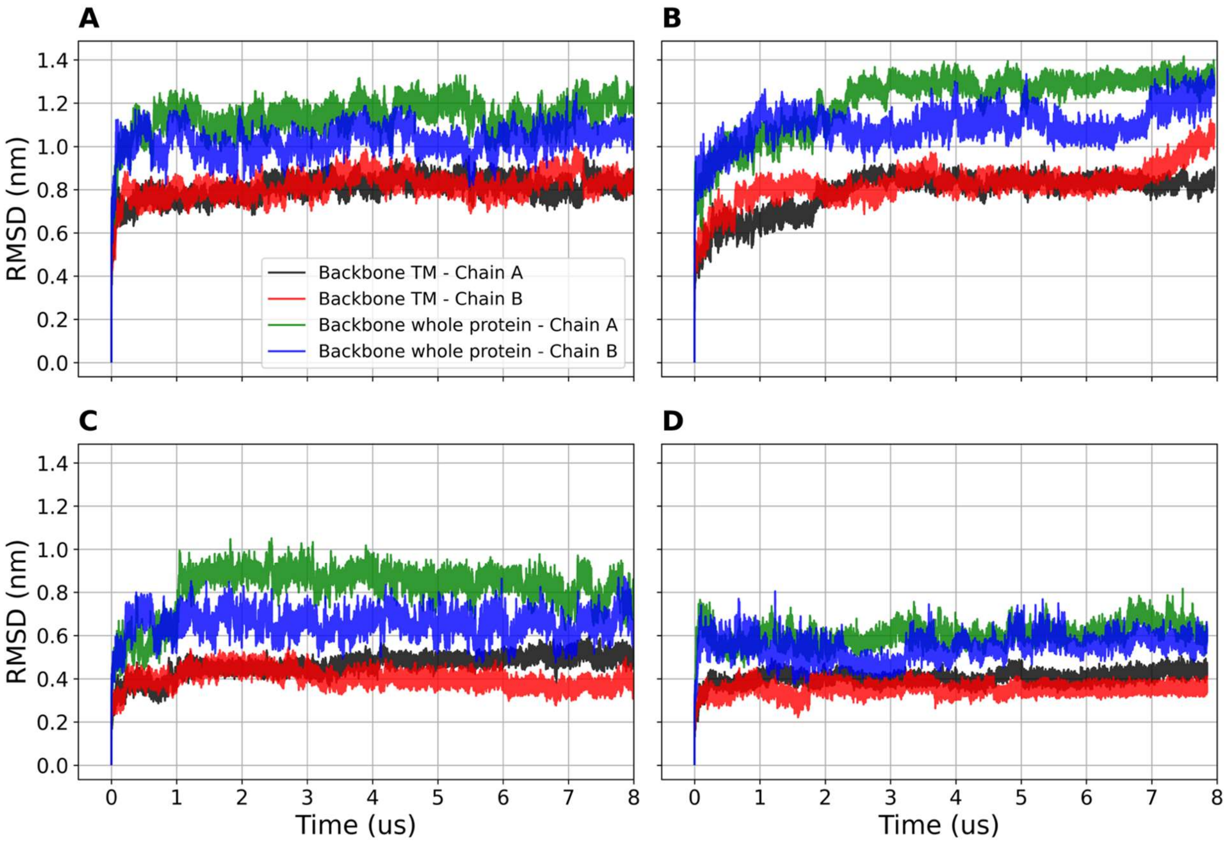

Figure S1. Backbone beads (for a definition of this, see [1]) RMSD of the whole protein and TM regions in **(A)** OMM and (**B**) chl_mem for *m*TSPO and in **(C)** OMM and (**D**) chl_mem for *m*TSPO(Rs). The RMSDs are computed with respect to the initial structures in this and all the other figures presented here.

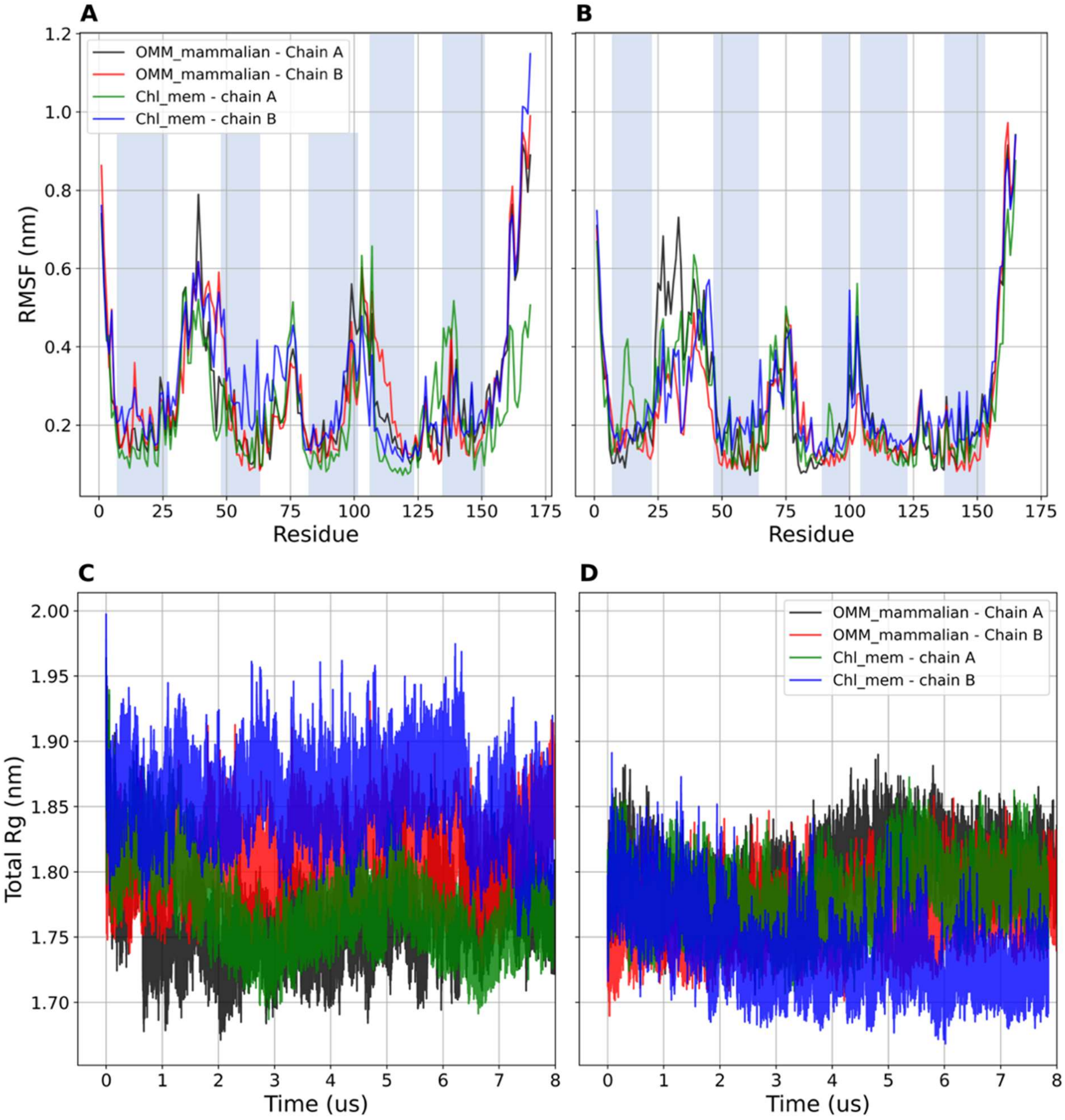

Figure S2. (**A-B**): RMSF values of each residue in **(A)** *m*TSPO and **(B)** *m*TSPO(Rs) embedded in both OMM and chl_mem. The shaded blue regions correspond to the five TMs. The analysis was performed on the equilibrated part of the trajectory, that is for the last 6 μs. (**C-D):** The radius of gyration (Rg) is plotted as a function of simulated time for **(C)** *m*TSPO and **(D)** *m*TSPO(Rs) embedded in both OMM and chl_mem.

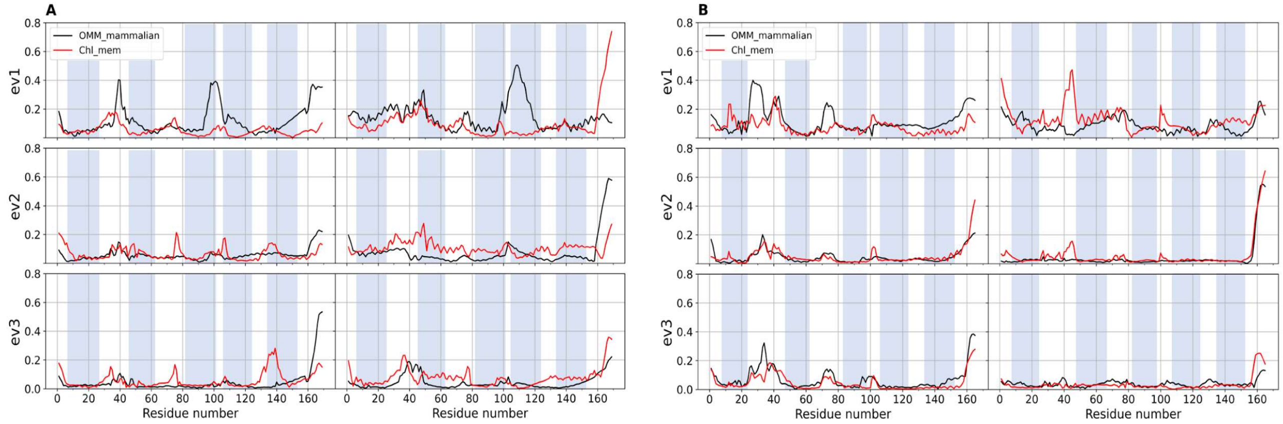

Figure S3. Projection of the backbone beads trajectory along the first three eigenvectors for both chains of **(A)** *m*TSPO and **(B)** *m*TSPO(Rs) dimeric structures. The shaded blue regions correspond to the five TMs. The analysis was performed on the equilibrated part of the trajectory, that is for the last 6 μs.

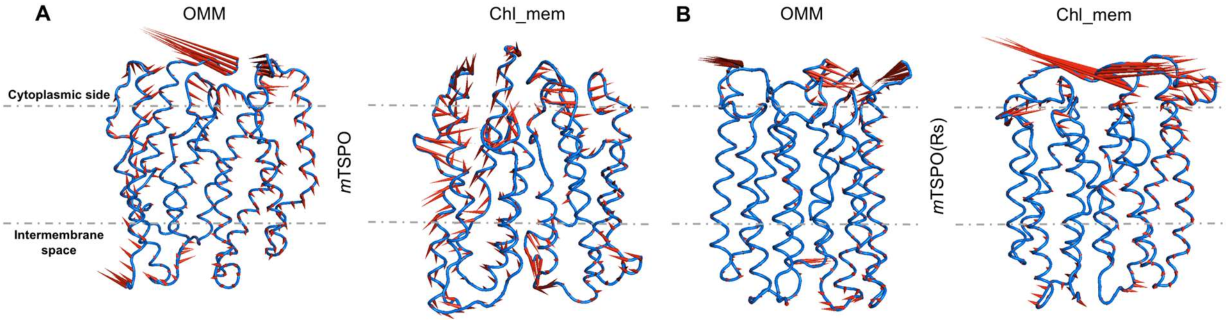

Figure S4. Porcupine plots depicting prominent motions averaged across the second normal mode for: **(A)** *m*TSPO, **(B)** *m*TSPO(Rs) in both OMM and chl_mem. A dotted line marks the approximate location of the membrane. Longer spines indicate the region with higher fluctuations for the second normal mode. These correspond to the cytoplasmic loops. The analysis was performed on the equilibrated trajectory, namely for the last 6 μs.

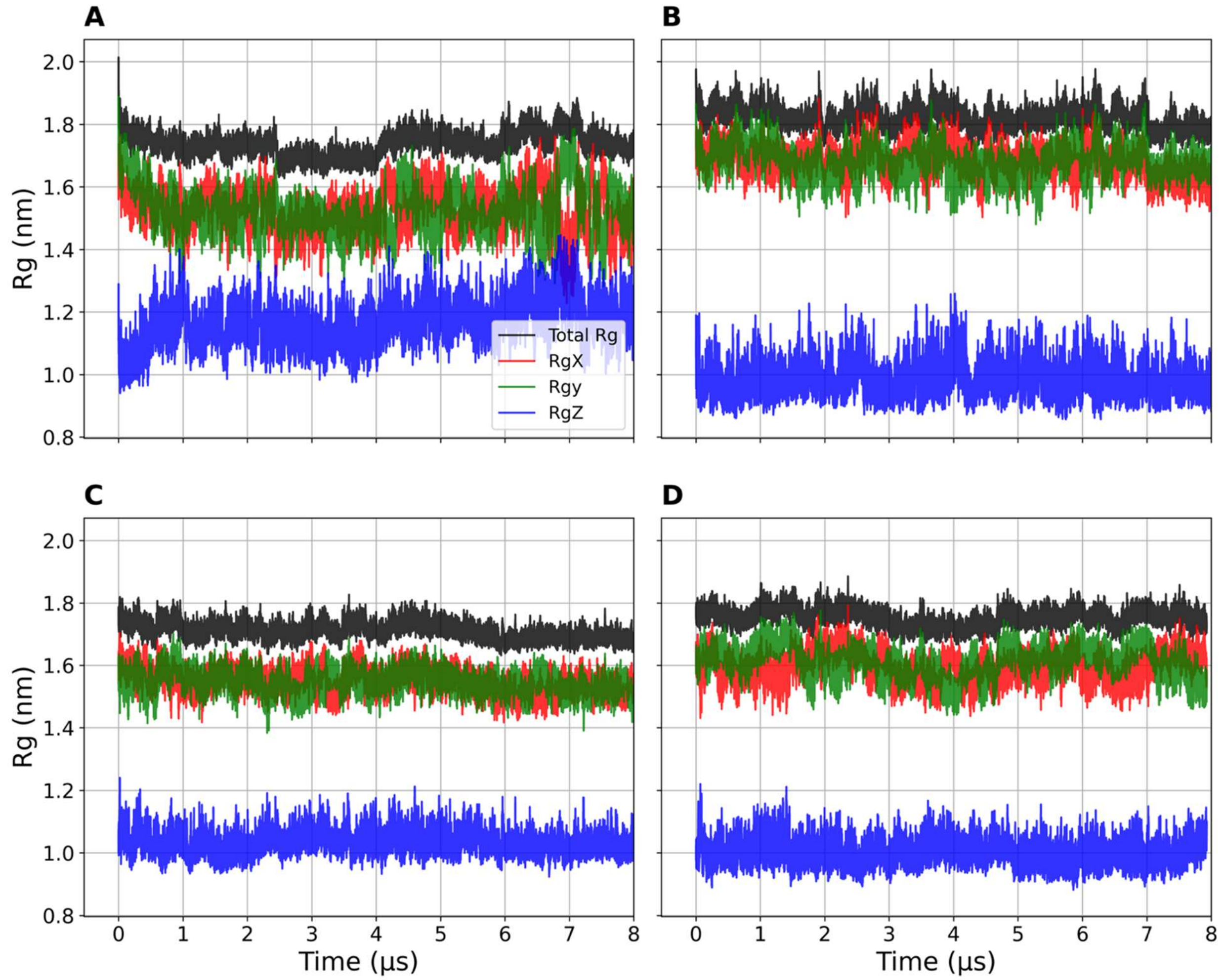

Figure S5. Radius of gyration (Rg) plotted as a function of the simulation time for *m*TSPO_mon in **(A)** OMM and **(B)** chl_mem, as well as for *m*TSPO(Rs)_mon in **(C)** OMM and **(D)** Chl_mem.

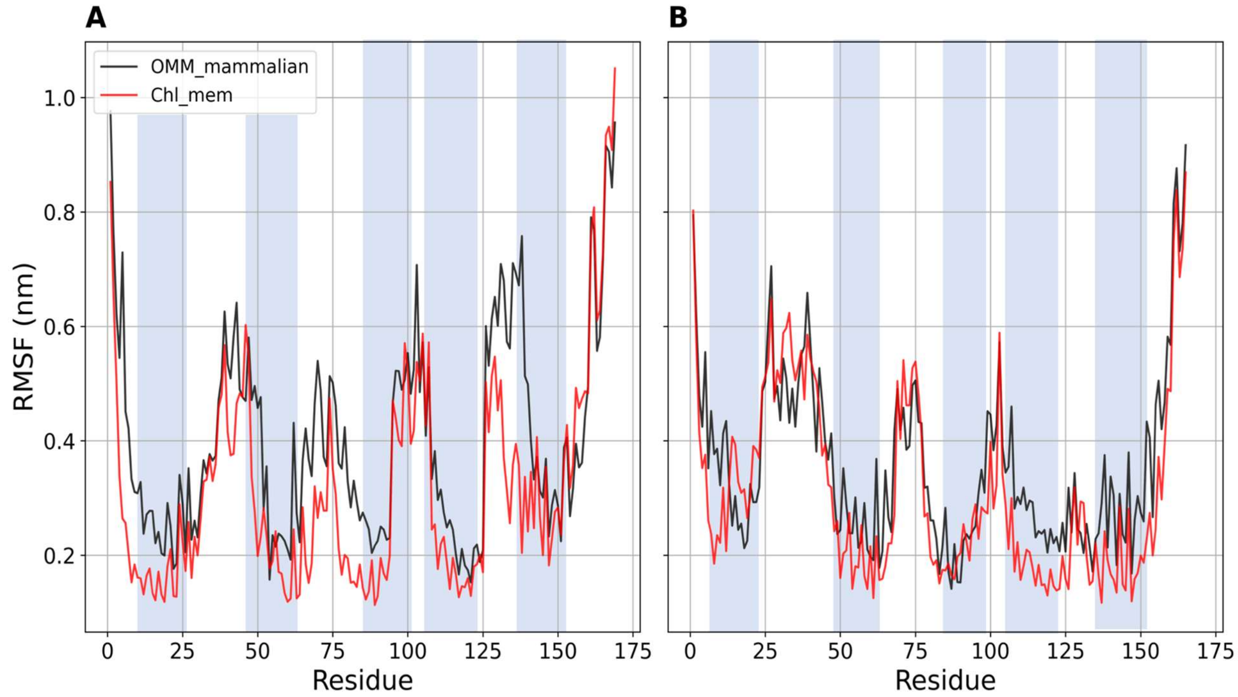

Figure S6. RMSF plots of **(A)** *m*TSPO_mon and **(B)** *m*TSPO(Rs)_mon in OMM and chl_mem. The shadowed blue regions correspond to the TMs. The analysis was performed on the equilibrated trajectory, that is for the last 6 μs.

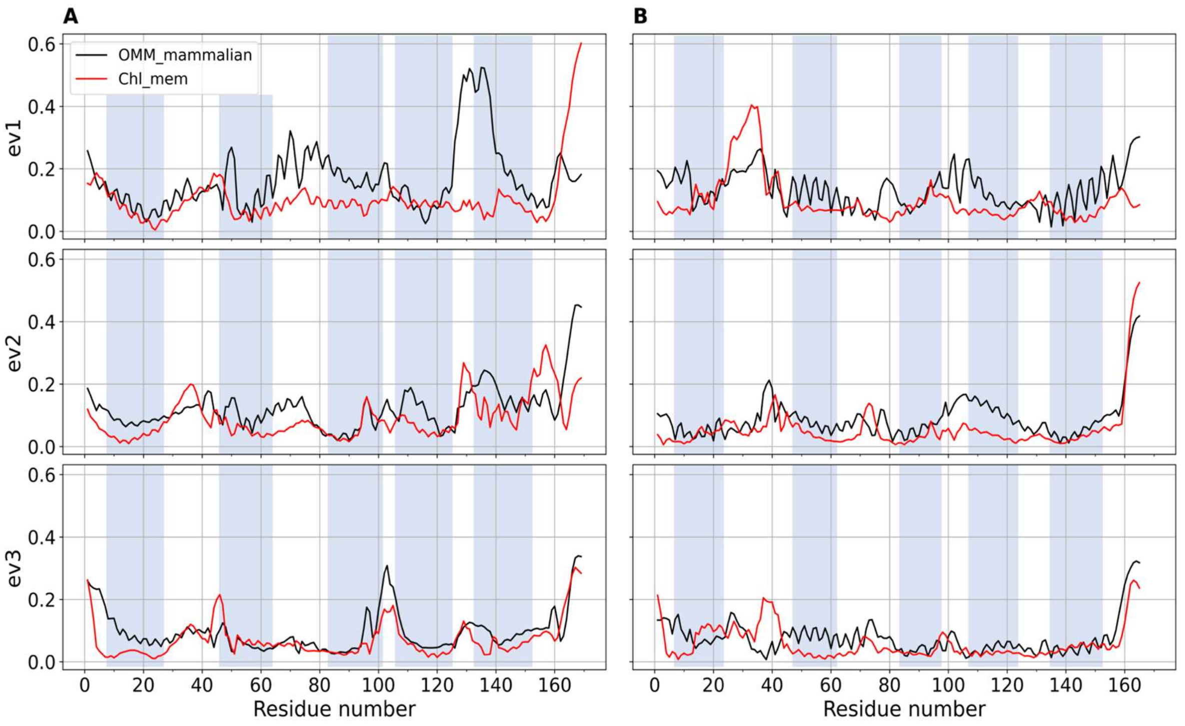

Figure S7. Projection of the backbone beads trajectory along the first three eigenvectors for **(A)** *m*TSPO_mon and **(B)** *m*TSPO(Rs)_mon. The shaded blue regions correspond to the five TMs. The analysis was performed on the equilibrated part of the trajectory, that is for the last 6 μs.

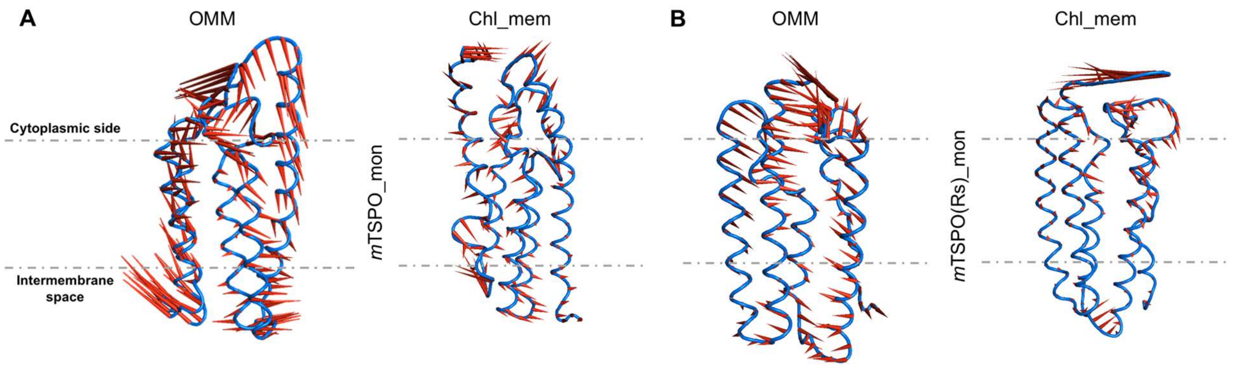

Figure S8. Porcupine plots depicting prominent motions averaged across the second normal mode for **(A)** *m*TSPO_mon and **(B)** *m*TSPO(Rs)_mon, in both OMM and chl_mem. A dotted line marks the approximate location of the membrane. Longer spines indicate the region with higher fluctuations for the second normal mode. These correspond to the cytoplasmic loops. The analysis was performed on the equilibrated trajectory, that is for the last 6 μs.

Bacterial Dimers

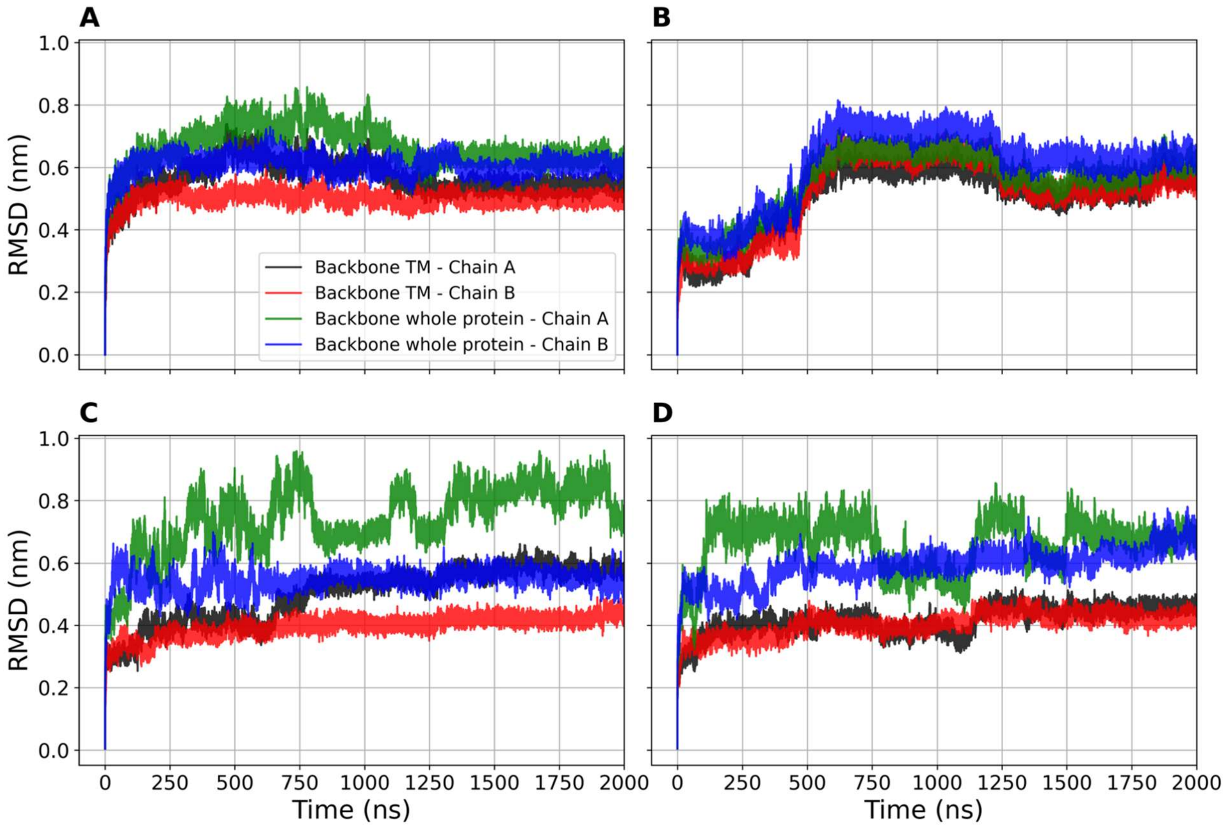

Figure S9. Backbone beads RMSD of the whole protein and TM regions for *Bc*TSPO in **(A)** Bc_mem and **(B)** chl_mem, as well as for *Rs*TSPO in **(C)** Rs_mem and **(D)** in chl_mem, plotted as a function of the simulation time.

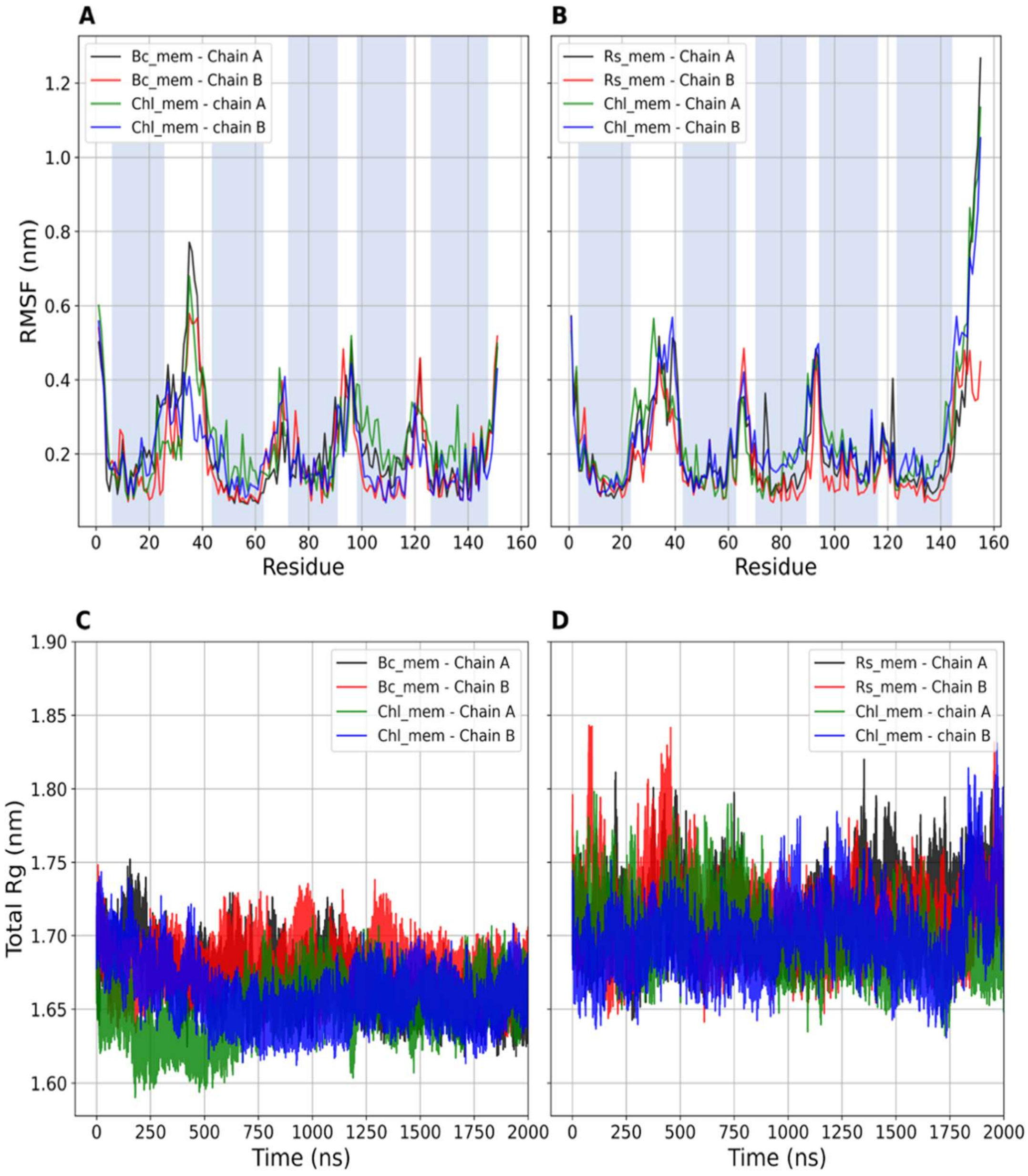

Figure S10. **(A-B):** RMSF values for each residue in **(A)** *Bc*TSPO in Bc_mem and chl_mem and **(B)** *Rs*TSPO in Rs_mem and chl_mem. **(C-D):** Radius of gyration (Rg) for (**C**) *Bc*TSPO in Bc_mem and chl_mem and (**D**) *Rs*TSPO in Rs_mem and chl_mem, plotted as a function of the simulated time. RMSF calculations were performed on the equilibrated part of the trajectories that is, for the last 1.3 μs.

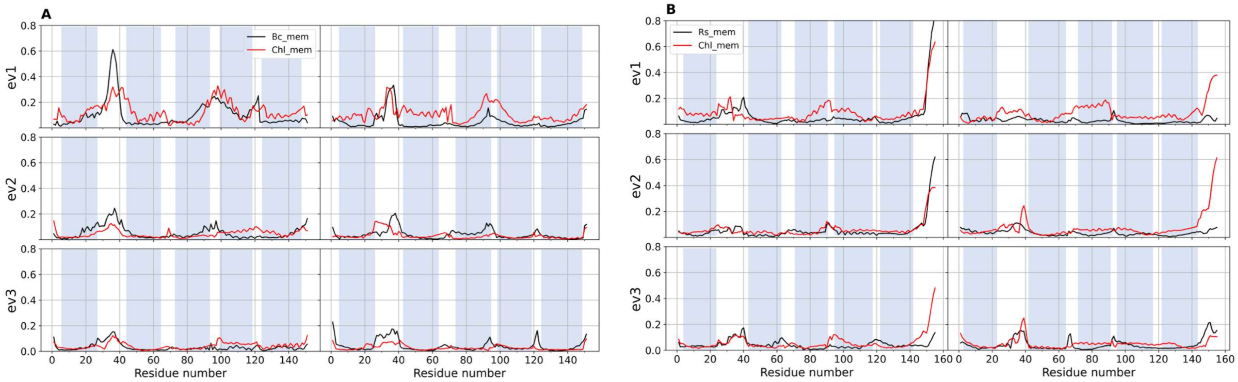

Figure S11. Projection of the backbone beads trajectory along the first three eigenvectors for **(A)** *Bc*TSPO and **(B)** *Rs*TSPO. The blue regions indicate the position of the TMs. The analysis was performed on the equilibrated part of the trajectory, that is, the last 1.3 μs.

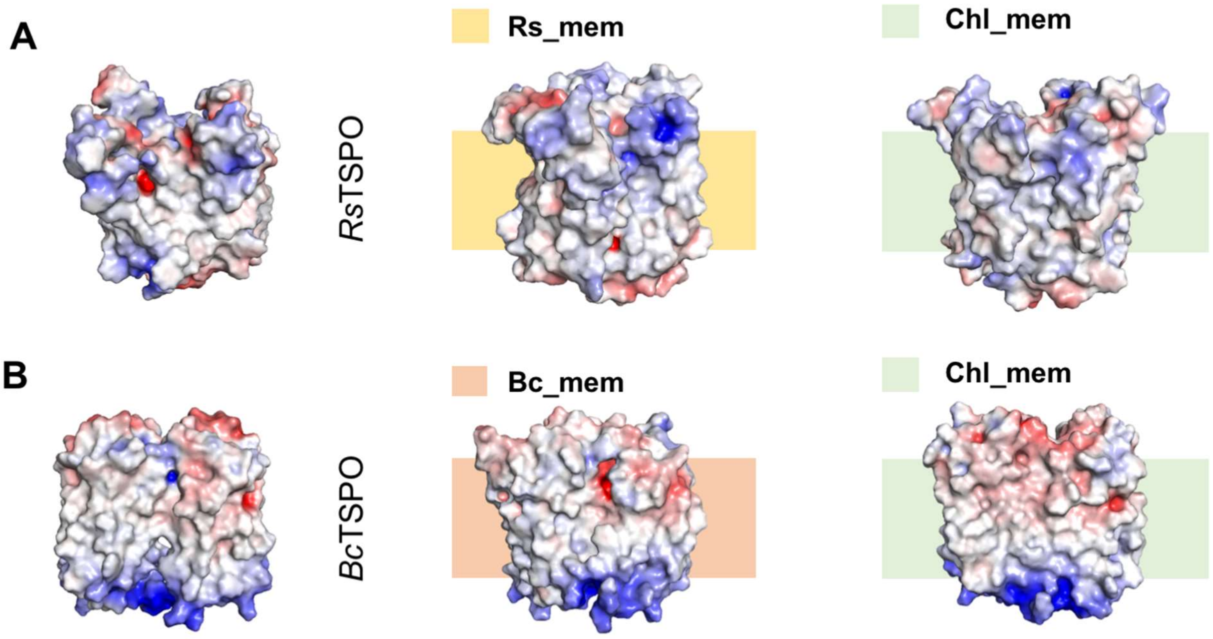

Figure S12. Electrostatic surface potentials in the initial and final MD structures for **(A)** *Rs*TSPO and **(B)** *Bc*TSPO embedded in Rs_mem/Bc_mem respectively and chl_mem. The MD structures were backmapped to all-atom resolution using the backward.py script [2]. The red and blue surfaces represent negative and positive electrostatic potentials, respectively. The maximum values of the potentials are -5 kT/e, +5kT/e, respectively.

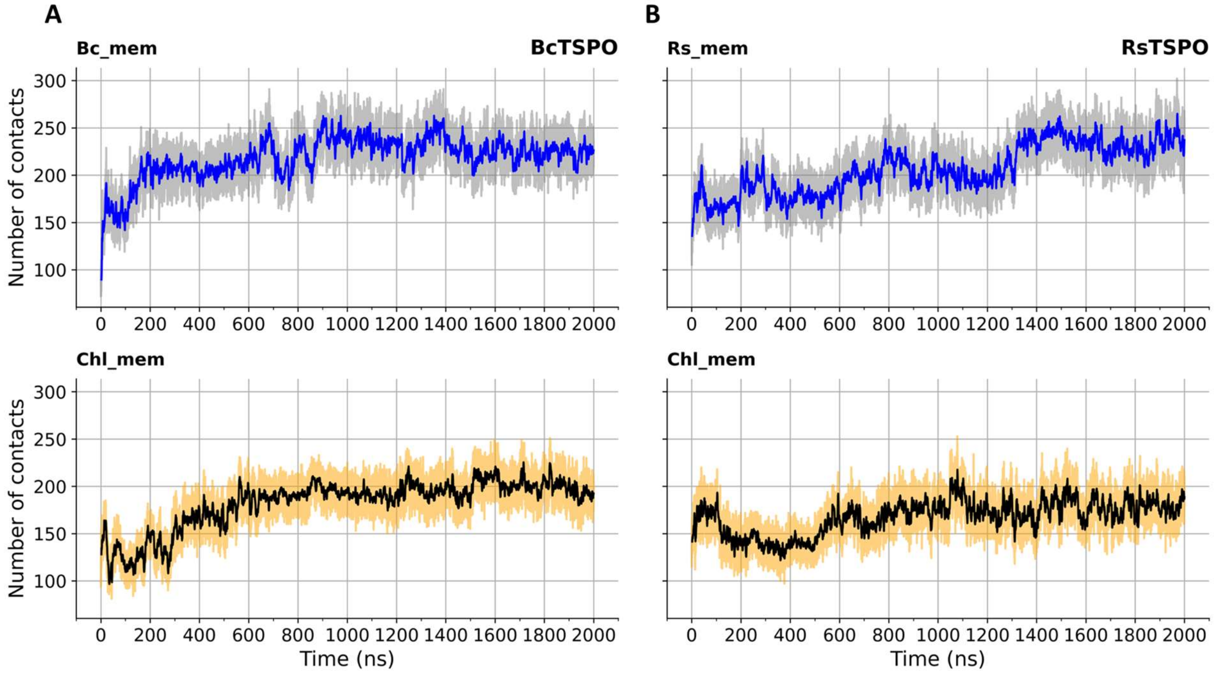

Figure S13. Number of subunit-subunit contact beads plotted as a function of the simulation time for **(A)** *Bc*TSPO in Bc_mem and chl_mem and (**B**) *Rs*TSPO in Rs_mem and chl_mem. For the definition of the contacts, see Methods Section in the main text.

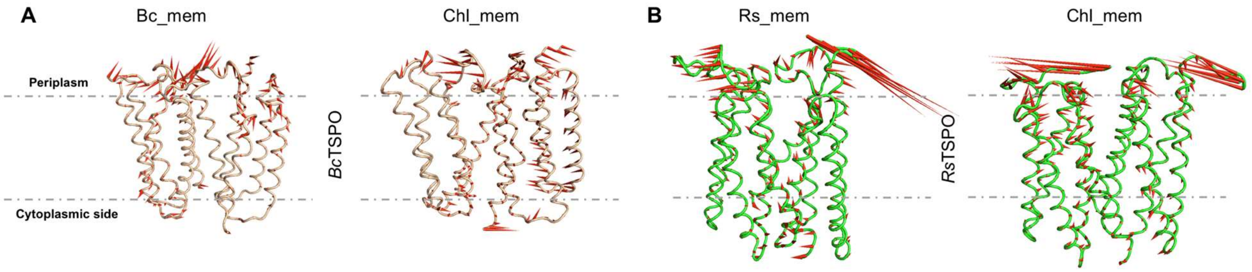

Figure S14. Porcupine plots depicting large scale motions averaged across the second normal mode for **(A)** *Bc*TSPO embedded in Bc_mem and chl_mem and **(B)** *Rs*TSPO embedded in Rs_mem and chl_mem. A dotted line marks the approximate location of the membrane. Longer spines indicate the region with higher fluctuations for the second normal mode. These correspond to the periplasm loops. The analysis was performed on the equilibrated trajectory, that is for the last 1.3 μs.

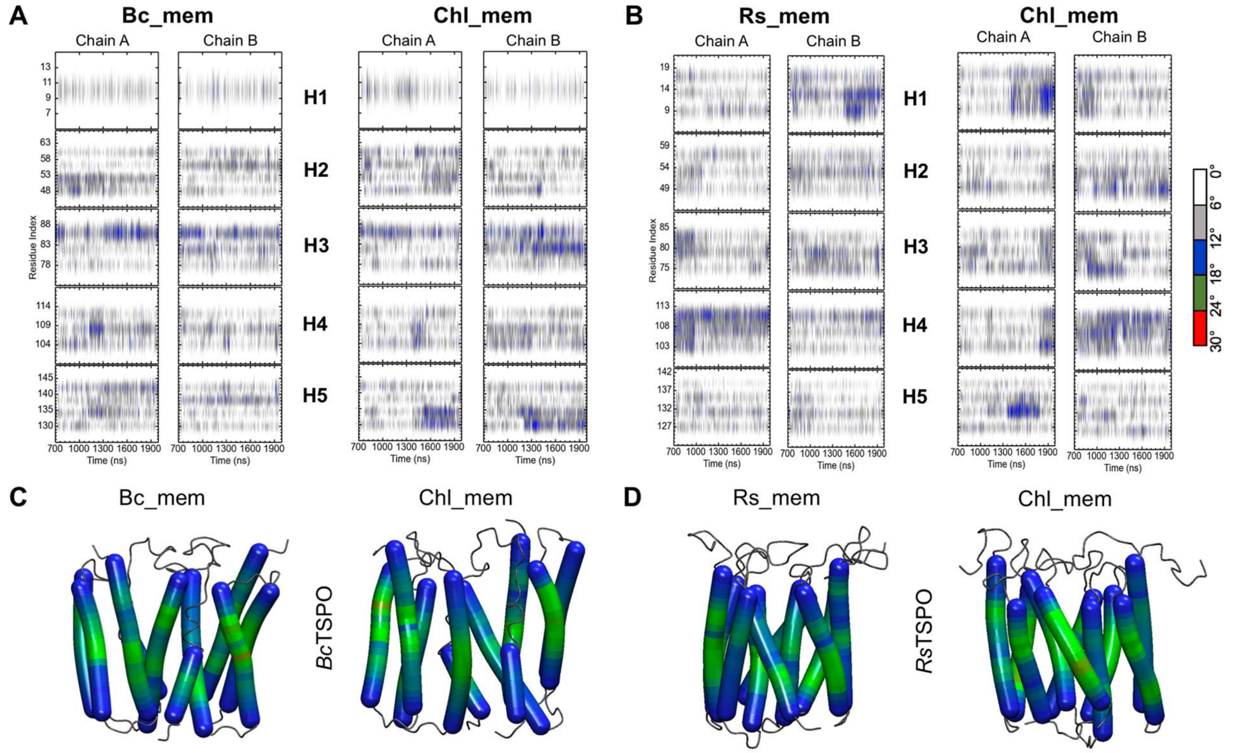

Figure S15. Helix bending of *Bc*TSPO embedded in **(A)** Bc_mem, chl_mem and for *Rs*TSPO in **(B)** Rs_mem and chl_mem, plotted as a function of the simulation time. The corresponding helix "hinge" regions are also shown on *Bc*TSPO (C) and *Rs*TSPO (D) structures in Bc_mem, Rs_mem and chl_mem: the highly bent regions are highlighted in red color, while the least and moderately bent regions are highlighted in blue and green colors, respectively. The analysis was performed on the equilibrated part of the trajectories, that is, the last 1.3 μs.

References

1. de Jong, D.H.; Singh, G.; Bennett, W.D.; Arnarez, C.; Wassenaar, T.A.; Schäfer, L.V.; Periole, X.; Tieleman, D.P.; Marrink, S.J. Improved parameters for the martini coarse-grained protein force field. Journal of chemical theory and computation 2013, 9, 687-697.
2. Wassenaar, T.A.; Pluhackova, K.; Böckmann, R.A.; Marrink, S.J.; Tieleman, D.P. Going backward: a flexible geometric approach to reverse transformation from coarse grained to atomistic models. Journal of chemical theory and computation 2014, 10, 676-690.
